# Supplementary material for: Loss of Gap Junction Delta-2 (GJD2) gene orthologs leads to refractive error in zebrafish
Source: Commun Biol. 2021 Jun 3;4:676. doi: 10.1038/s42003-021-02185-z (PMC8175550; doi:10.1038/s42003-021-02185-z)
Supplement: Supplementary file 2 — Supplementary Information [file 42003_2021_2185_MOESM2_ESM.pdf]

Supplementary Information

Loss of Gap Junction Delta-2 (*GJD2*) gene orthologs leads to refractive error in zebrafish.

Wim H. Quint[1,2*], Kirke C.D. Tadema[1,2], Erik de Vrieze[3], Rachel M. Lukowicz[4], Sanne Broekman[3], Beerend H.J. Winkelman[1,5], Melanie Hoevenaars[1,2], H. Martijn de Gruiter[6], Erwin van Wijk[3], Frank Schaeffel[7,8], Magda Meester-Smoor[1,9], Adam C. Miller[4], Rob Willemsen[2], Caroline C.W. Klaver[1,8,9,10], Adriana I. Iglesias[1,2*]

[1]Department of Ophthalmology, Erasmus Medical Center, Rotterdam, The Netherlands
[2]Department of Clinical Genetics, Erasmus Medical Center, Rotterdam, The Netherlands
[3]Department of Otorhinolaryngology, Donders Institute for Brain, Cognition and Behavior, Radboud University Medical Center, Nijmegen, Netherlands
[4]Institute of Neuroscience, University of Oregon, Eugene, United States
[5]Department of Cerebellar Coordination and Cognition, Netherlands Institute for Neuroscience, Amsterdam, The Netherlands
[6]Optical Imaging Centre, Erasmus Medical Center, Rotterdam, The Netherlands
[7]Institute for Ophthalmic Research, University of Tübingen, Tübingen, Germany
[8]Institute of Molecular and Clinical Ophthalmology Basel, Basel, Switzerland
[9]Department of Epidemiology, Erasmus Medical Center, Rotterdam, The Netherlands
[10]Department of Ophthalmology, Radboud University Medical Center, Nijmegen, The Netherlands

Corresponding authors: Adriana I. Iglesias (a.iglesiasgonzalez@erasmusmc.nl), Wim H. Quint (w.quint@erasmusmc.nl)

Supplementary Material

Supplementary tables

Supplementary Table S1. Layer specific immunoreactivity of anti-pan-Cx35 and anti-Cx35.5 (*gjd2a*).

	anti-pan-Cx35 (Cx35.1 and Cx35.5)				anti-Cx35.5 (*gjd2a*)			
	PR	OPL	IPL	GCL	PR	OPL	IPL	GCL
WT Control	+	+	+	+	+	+	+	+
gjd2a (Cx35.5) mutant	+	+	+	+	-	-	-	-
gjd2b (Cx35.1) mutant	+	+	+	+				
gjd2a gjd2b double mutant	-	-	-	-	-	-	-	-

PR: photoreceptors, OPL: outer plexiform layer, IPL: inner plexiform layer, GCL: ganglion cell layer.

Supplementary Figures

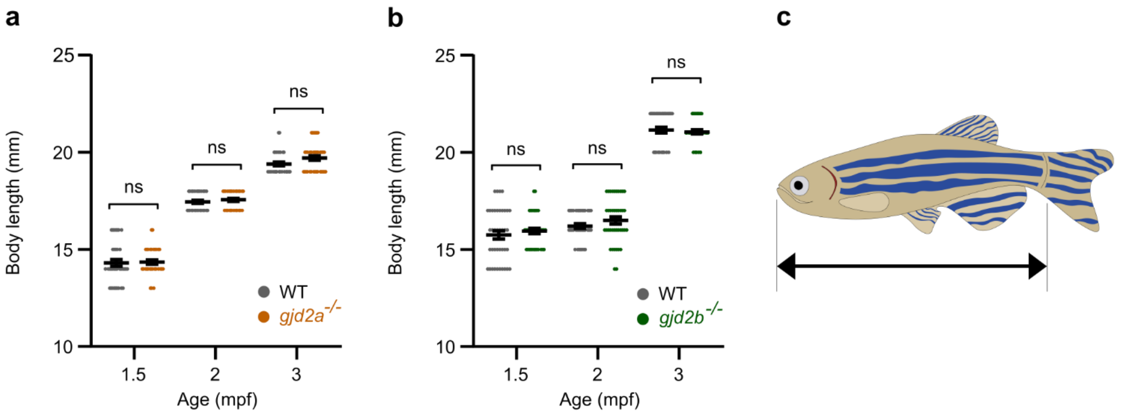

Supplementary Figure S1. Biometric alterations were independent of body length. Rounded body lengths of *gjd2a* (Cx35.5) (**a**) and *gjd2b* (Cx35.1) (**b**) mutants. During SD-OCT measurements, both fish lines were compared with size-matched WT controls. Sample size: n=40 eyes. Scale bars: SEM. Significance: ns = not significant, *p < 0.05, **p < 0.01. ***p < 0 .001. **c** Bodylength was measured from the tip of the head to the proximal end of the caudal fin.

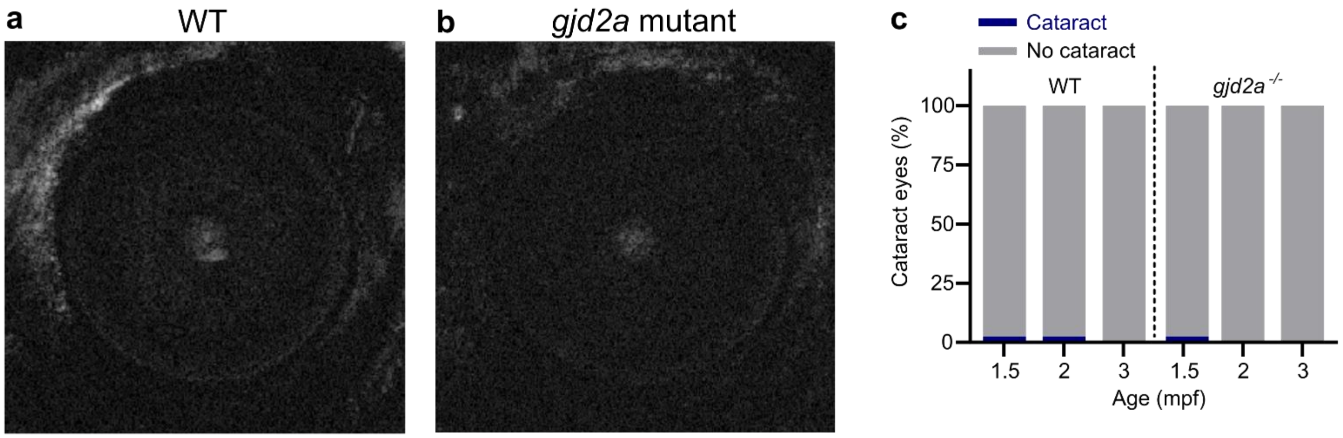

Supplementary Figure S2. Quantification of cataract in 1-3mpf *gjd2a* (Cx35.5) mutant fish.
a, b Coronal SD-OCT sections of typical 3mpf lenses of WT control (**a**) and *gjd2a* (Cx35.5) mutant (**b**) fish. **c** Proportion of cataractous lenses in 1.5mpf, 2mpf, and 3mpf SD-OCT data (n=40 eyes) indicating that *gjd2a* (Cx35.5) mutants showed no cataract development during these stages of development.

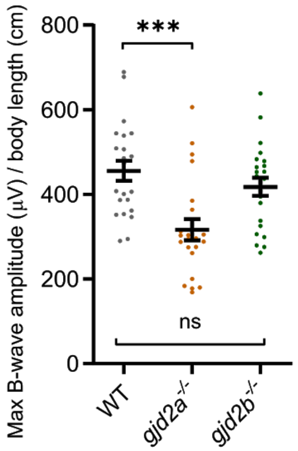

Supplementary Figure S3. ERG normalized by body length.
Maximum B-wave amplitude response normalized by body length. Scale bars: SEM. Significance: ns = not significant, *p < 0.05, **p < 0.01. ***p<0.001.

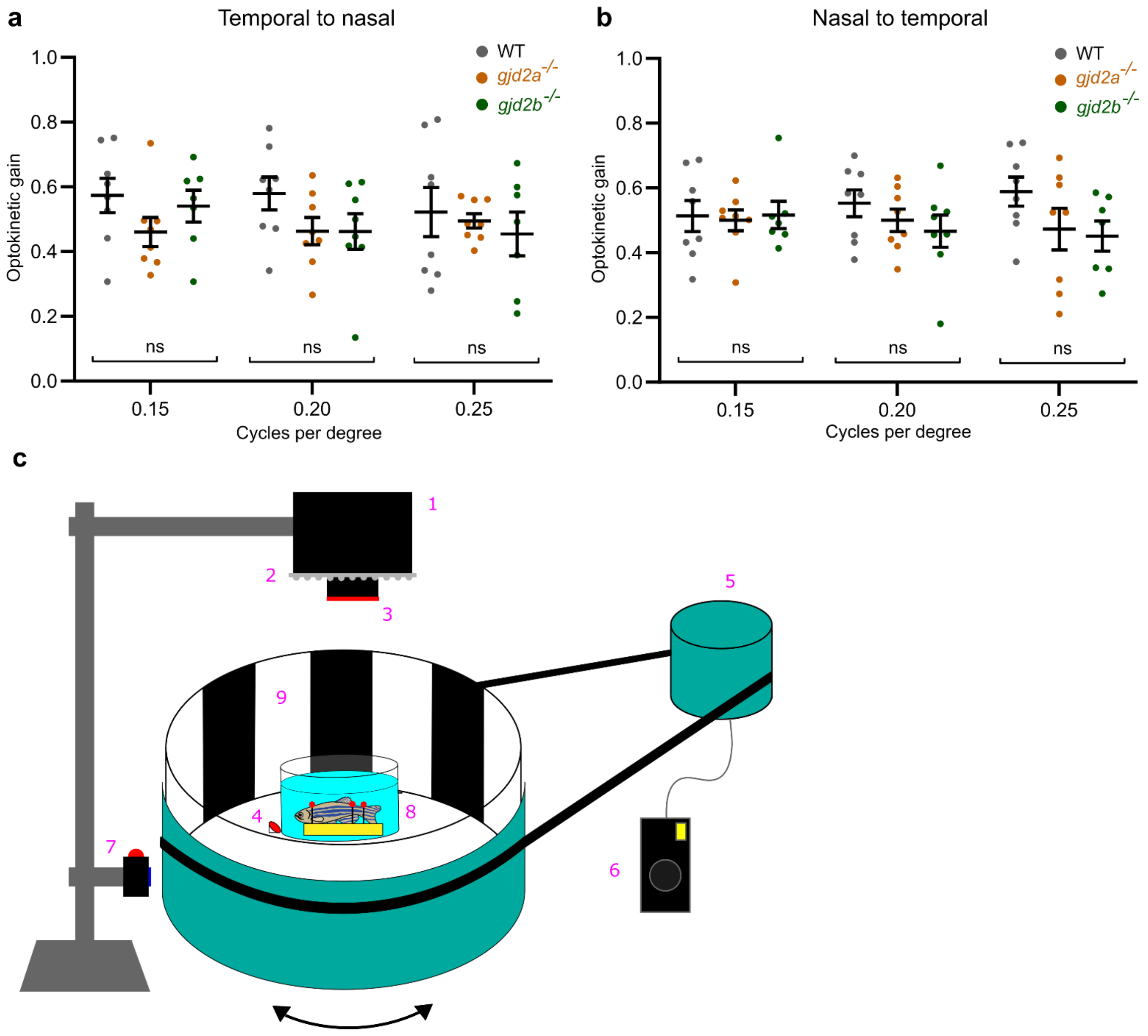

Supplementary Figure S4. Optokinetic gain.

Optokinetic gain in temporal to nasal (**a**) and nasal to temporal (**b**) direction for spatial frequencies ranging from 0.15-0.25cpd. **c** OKR device for the use in adult zebrafish. 1: camera, 2: circular led array for drum lightning with visible light, 3: long pass filter, 4: infrared LED illuminator, 5: motor, 6: velocity control, 7: tachometer, 8: fish aquarium with fixation device, 9: drum. Scale bars: SEM. Significance: ns = not significant, $*p < 0.05$, $**p < 0.01$. $***p<0.001$. Cpd: cycles per degree.

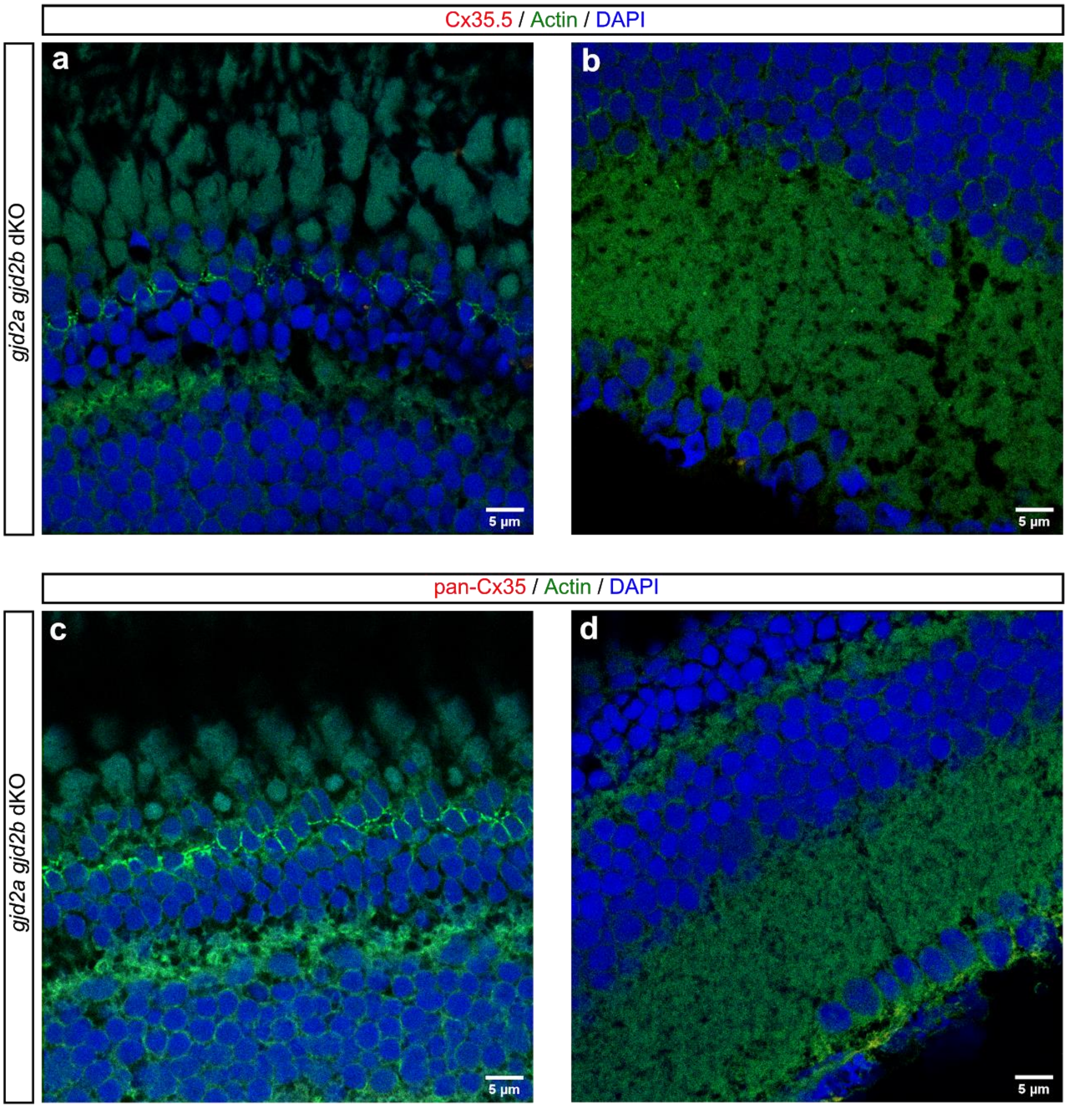

Supplementary Figure S5. Supplementary immunostainings retina.
Negative immunostaining for anti-Cx35.5 (*gjd2a*) (**a, b**) and anti-pan-Cx35 (red) in the *gjd2a; gjd2b* double mutant (**a-d**). DAPI (blue) and anti-actin (green) are used for orientation.

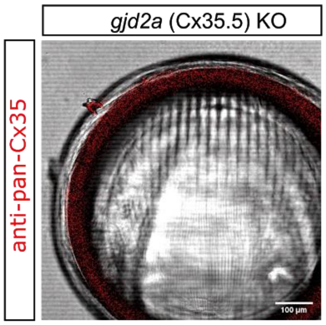

Supplementary Figure S6. Supplementary immunostainings lens.
Anti-pan-Cx35 (red) immunostaining in isolated 6mpf Cx35.5 (*gjd2a*) mutant lenses showing a signal at the outer cortical layer of the lens.

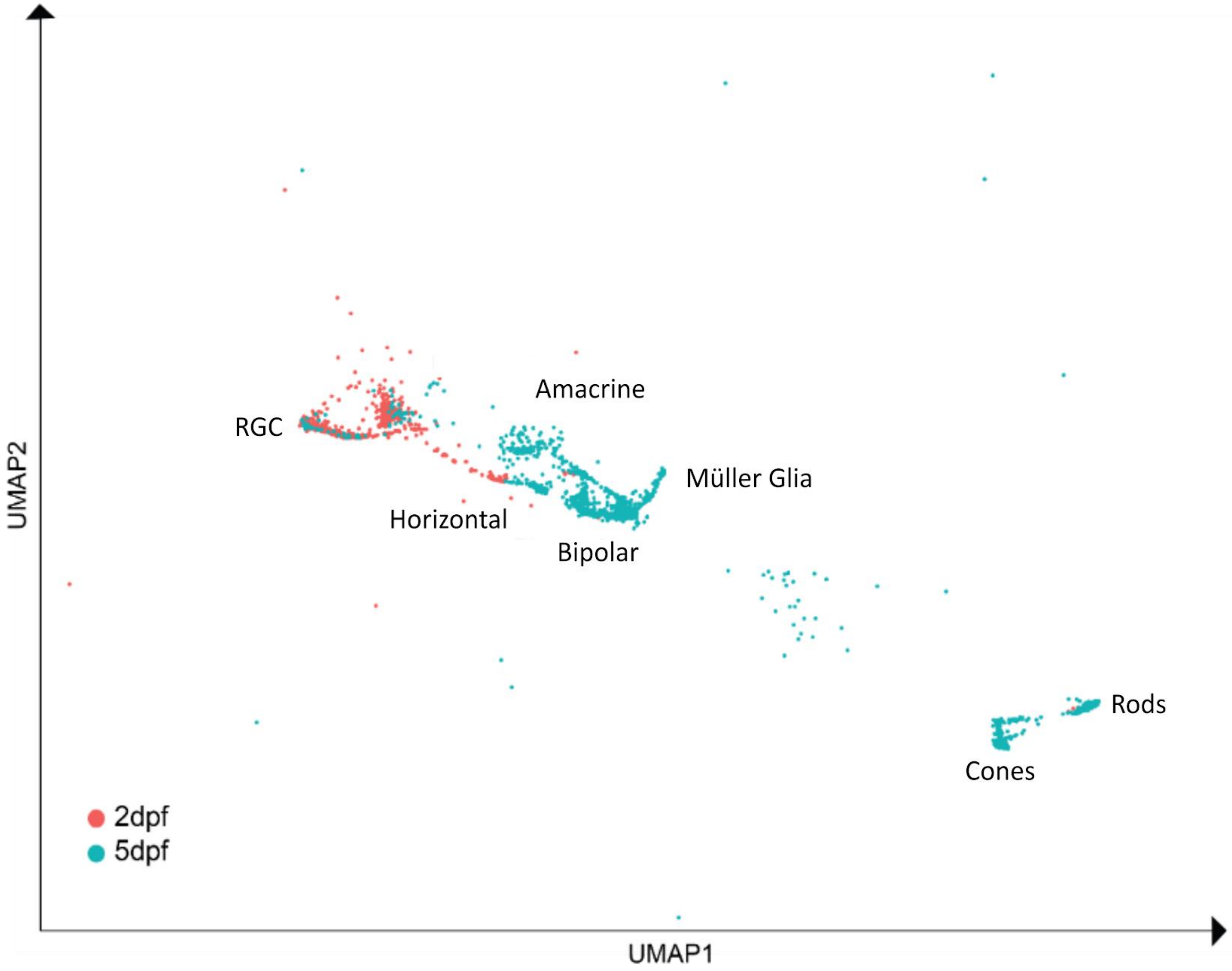

Supplementary Figure S7. Age of origin of cells in scRNAseq clusters.
Data were aggregated from embryos sampled at 2dpf (pink) and 5dpf (cyan). Some bioinformatically-determined clusters contain cells from both times of isolation, while most are exclusive to 5dpf. Dpf: days post fertilization, UMAP: Uniform Manifold Approximation and Projection, RGC: retinal ganglion cell.
