# Supplementary material for: Loss of Gap Junction Delta-2 (GJD2) gene orthologs leads to refractive error in zebrafish
Source: Commun Biol. 2021 Jun 3;4:676. doi: 10.1038/s42003-021-02185-z (PMC8175550; doi:10.1038/s42003-021-02185-z)
Supplement: Supplementary file 3 — Description of Supplementary Files [file 42003_2021_2185_MOESM3_ESM.pdf]

Description of Additional Supplementary Files

File name: Supplementary Data S1
Description: *gjd2a* (Cx35.5) mutant at 1.5-3mpf
Caption: AL: axial length; ACD: anterior chamber depth; VCD: vitreous chamber depth; RPE: retinal pigmented epithelium; mpf: months post-fertilization; SEM: standard error of the mean; β-coefficient: unstandardized coefficient, representing the effect of genotype on the ocular axial length, SE: standard error. Effect (%): β-coefficient divided by the mean WT metrics.

File name: Supplementary Data S2
Description: *gjd2b* (Cx35.1) mutant at 1.5-3mpf
Caption: AL: axial length; ACD: anterior chamber depth; VCD: vitreous chamber depth; RPE: retinal pigmented epithelium; mpf: months post-fertilization; SEM: standard error of the mean; β-coefficient: unstandardized coefficient, representing the effect of genotype on the ocular axial length, SE: standard error. Effect (%): β-coefficient divided by the mean WT metrics.

File name: Supplementary Data S3
Description: *gjd2b* (Cx35.1) mutant at 6mpf
Caption: AL: axial length; ACD: anterior chamber depth; VCD: vitreous chamber depth; RPE: retinal pigmented epithelium; mpf: months post-fertilization; SEM: standard error of the mean; β-coefficient: unstandardized coefficient, representing the effect of genotype on the ocular axial length, SE: standard error. Effect (%): β-coefficient divided by the mean WT metrics.

File name: Supplementary Data S4
Description: Top 16 most differential genes
Caption: Bold genes support annotation based on ZFIN and other pubs. RGC: retinal ganglion cell.
