# Supplementary material for: Loss of Gap Junction Delta-2 (GJD2) gene orthologs leads to refractive error in zebrafish
Source: Commun Biol. 2021 Jun 3;4:676. doi: 10.1038/s42003-021-02185-z (PMC8175550; doi:10.1038/s42003-021-02185-z)
Supplement: Supplementary file 8 — Reporting Summary [file 42003_2021_2185_MOESM8_ESM.pdf]

nature research

Corresponding author(s): W.H.Quint, A.I. Iglesias

Last updated by author(s): Mar 31, 2021

Reporting Summary

Nature Research wishes to improve the reproducibility of the work that we publish. This form provides structure for consistency and transparency in reporting. For further information on Nature Research policies, see our Editorial Policies and the Editorial Policy Checklist.

Statistics

For all statistical analyses, confirm that the following items are present in the figure legend, table legend, main text, or Methods section.

n/a	Confirmed	
☐	☒	The exact sample size (*n*) for each experimental group/condition, given as a discrete number and unit of measurement
☐	☒	A statement on whether measurements were taken from distinct samples or whether the same sample was measured repeatedly
☐	☒	The statistical test(s) used AND whether they are one- or two-sided<br>*Only common tests should be described solely by name; describe more complex techniques in the Methods section.*
☐	☒	A description of all covariates tested
☐	☒	A description of any assumptions or corrections, such as tests of normality and adjustment for multiple comparisons
☐	☒	A full description of the statistical parameters including central tendency (e.g. means) or other basic estimates (e.g. regression coefficient) AND variation (e.g. standard deviation) or associated estimates of uncertainty (e.g. confidence intervals)
☐	☒	For null hypothesis testing, the test statistic (e.g. *F*, *t*, *r*) with confidence intervals, effect sizes, degrees of freedom and *P* value noted<br>*Give P values as exact values whenever suitable.*
☒	☐	For Bayesian analysis, information on the choice of priors and Markov chain Monte Carlo settings
☒	☐	For hierarchical and complex designs, identification of the appropriate level for tests and full reporting of outcomes
☐	☒	Estimates of effect sizes (e.g. Cohen's *d*, Pearson's *r*), indicating how they were calculated

Our web collection on statistics for biologists contains articles on many of the points above.

Software and code

Policy information about availability of computer code

Data collection	The OCT processing and analysis code (Matlab), the custom photorefraction (C++) and the OKR software (Python version 3.8) can be obtained from the corresponding author on request.
Data analysis	Matlab version R2020a, R version 3.6.2, Prism version 8.4.1, Python version 3.8.

For manuscripts utilizing custom algorithms or software that are central to the research but not yet described in published literature, software must be made available to editors and reviewers. We strongly encourage code deposition in a community repository (e.g. GitHub). See the Nature Research guidelines for submitting code & software for further information.

Data

Policy information about availability of data

All manuscripts must include a data availability statement. This statement should provide the following information, where applicable:

- Accession codes, unique identifiers, or web links for publicly available datasets
- A list of figures that have associated raw data
- A description of any restrictions on data availability

All data generated or analyzed during this study are included in this published article and its supplementary information files. Any additional (raw) data are available from the corresponding author on request. Data used in the scRNA-seq study is publicly available at 'https://www.adammillerlab.com/resources-1' and 'http://cells.ucsc.edu/?ds=zebrafish-dev'.

Field-specific reporting

Please select the one below that is the best fit for your research. If you are not sure, read the appropriate sections before making your selection.

- [x] Life sciences
- [ ] Behavioural & social sciences
- [ ] Ecological, evolutionary & environmental sciences

For a reference copy of the document with all sections, see nature.com/documents/nr-reporting-summary-flat.pdf

Life sciences study design

All studies must disclose on these points even when the disclosure is negative.

Sample size	Power analysis of eye size measurements (SD-OCT). It assumes a minimum difference in ocular axial length of 10 µm (~2.5 diopters either towards myopia or hyperopia) and a standard deviation of 15 µm, yielding a medium effect size of 0.6. Given an effect size of 0.6, n=40 measurements per group are required to achieve statistical significance at the 5% level.<br>Sample size of the electroretinogram (ERG) study is n=22 fish, and of the optokinetic response (OKR) assesment n=10 fish. This sample size is comparable with other studies (e.g., Dona et al., Exp Eye Research 2018, Deveau et al., Plos Genetics 2020)
Data exclusions	No data were excluded from the analysis.
Replication	In the gjd2a mutant, OCT findings were confirmed by photorefraction. In the gjd2b mutant, we used multiple techniques (i.e., SD-OCT, differential interference contrast (DIC) microscopy and light propagation studies to confirm the presence of a cataractous phenotype, showing robustness of our findings. We did not perform a formal replication study.
Randomization	No randomization was performed
Blinding	No blinding was performed

Reporting for specific materials, systems and methods

We require information from authors about some types of materials, experimental systems and methods used in many studies. Here, indicate whether each material, system or method listed is relevant to your study. If you are not sure if a list item applies to your research, read the appropriate section before selecting a response.

Materials & experimental systems

n/a	Involved in the study	
[ ]	[x]	Antibodies
[x]	[ ]	Eukaryotic cell lines
[x]	[ ]	Palaeontology and archaeology
[ ]	[x]	Animals and other organisms
[x]	[ ]	Human research participants
[x]	[ ]	Clinical data
[x]	[ ]	Dual use research of concern

Methods

n/a	Involved in the study	
[x]	[ ]	ChIP-seq
[x]	[ ]	Flow cytometry
[x]	[ ]	MRI-based neuroimaging

Antibodies

Antibodies used	MAB3045: Merck/Millipore, clone 8F6.2<br><br>Anti-gjd2a/Cx35.5: generated and described by Miller et al. (Miller, A. C. et al. A genetic basis for molecular asymmetry at vertebrate electrical synapses. eLife vol. 6 (2017))<br><br>Anti-mouse-cy5: Jackson ImmunoResearch, Cat. Nr. 715-176-150<br><br>Anti-rabbit-cy5, Jackson ImmunoResearch, Cat. Nr. 711-175-152<br><br>Phalloidin546, ThermoFisher, Cat. Nr. A22283
Validation	MAB3045:<br>- O'Brien, J., Nguyen, H. B. & Mills, S. L. Cone photoreceptors in bass retina use two connexins to mediate electrical coupling. J. Neurosci. 24, 5632–5642 (2004).<br>50.<br>- Pereda, A. et al. Connexin35 Mediates Electrical Transmission at Mixed Synapses on Mauthner Cells. The Journal of Neuroscience vol. 23 7489–7503 (2003).

	Anti-gjd2a/Cx35.5: Miller, A. C. et al. A genetic basis for molecular asymmetry at vertebrate electrical synapses. eLife vol. 6 (2017).

Animals and other organisms

Policy information about studies involving animals; ARRIVE guidelines recommended for reporting animal research

Laboratory animals	Danio Rerio (zebrafish); AB/Tuebingen; male and female; 1.5, 2, 6, and 9 mpf
Wild animals	No wild animals
Field-collected samples	The study did not involve samples collected in the field
Ethics oversight	All animals were raised and treated in accordance to the Dutch animal welfare legislation and the guidelines from the experimental animal health care center (EDC:Experimenteel Dier Centrum) of the Erasmus Medical Center Rotterdam, The Netherlands. All experiments were conducted in accordance with the European Commission Council Directive.

Note that full information on the approval of the study protocol must also be provided in the manuscript.
